# Comparative Temporal Transcriptome Profiling of Wheat near Isogenic Line Carrying *Lr*57 under Compatible and Incompatible Interactions

**DOI:** 10.3389/fpls.2016.01943

**Published:** 2016-12-23

**Authors:** Inderjit S. Yadav, Amandeep Sharma, Satinder Kaur, Natasha Nahar, Subhash C. Bhardwaj, Tilak R. Sharma, Parveen Chhuneja

**Affiliations:** ^1^School of Agricultural Biotechnology, Punjab Agricultural UniversityLudhiana, India; ^2^Regional Research Station, Indian Institute of Wheat and Barley ResearchFlowerdale, Shimla; ^3^National Research Centre on Plant BiotechnologyNew Delhi, India

**Keywords:** differential gene expression, *Lr57*, leaf rust, pathways, RNAseq, R-genes, transcriptomics, wheat

## Abstract

Leaf rust caused by *Puccinia triticina* (*Pt*) is one of the most important diseases of bread wheat globally. Recent advances in sequencing technologies have provided opportunities to analyse the complete transcriptomes of the host as well as pathogen for studying differential gene expression during infection. Pathogen induced differential gene expression was characterized in a near isogenic line carrying leaf rust resistance gene *Lr57* and susceptible recipient genotype WL711. RNA samples were collected at five different time points 0, 12, 24, 48, and 72 h post inoculation (HPI) with Pt 77-5. A total of 3020 transcripts were differentially expressed with 1458 and 2692 transcripts in WL711 and WL711+*Lr57*, respectively. The highest number of differentially expressed transcripts was detected at 12 HPI. Functional categorization using Blast2GO classified the genes into biological processes, molecular function and cellular components. WL711+*Lr57* showed much higher number of differentially expressed nucleotide binding and leucine rich repeat genes and expressed more protein kinases and pathogenesis related proteins such as chitinases, glucanases and other PR proteins as compared to susceptible genotype. Pathway annotation with KEGG categorized genes into 13 major classes with carbohydrate metabolism being the most prominent followed by amino acid, secondary metabolites, and nucleotide metabolism. Gene co-expression network analysis identified four and eight clusters of highly correlated genes in WL711 and WL711+*Lr57*, respectively. Comparative analysis of the differentially expressed transcripts led to the identification of some transcripts which were specifically expressed only in WL711+*Lr57*. It was apparent from the whole transcriptome sequencing that the resistance gene *Lr57* directed the expression of different genes involved in building the resistance response in the host to combat invading pathogen. The RNAseq data and differentially expressed transcripts identified in present study is a genomic resource which can be used for further studying the host pathogen interaction for *Lr57* and wheat transcriptome in general.

## Introduction

Leaf rust caused by *Puccinia triticina* (Pt) is one of the most important diseases of bread wheat (*Triticum aestivum*) causing significant yield losses in almost all the wheat growing regions globally. Leaf rust epidemics sometimes cause significant yield losses (Kolmer et al., 2007). About 60–70% infection on the flag leaf at spike emergence can cause 30% yield losses whereas infections at earlier plant stages can account to 50% yield losses (Huerta-Espino et al., 2011). Genetic resistance is the most efficient method for disease management. Much remains unknown regarding the molecular basis of disease development. Nevertheless, numerous genes involved in the wheat-*P. triticina* response have been identified. More than 76 genes for leaf rust have been cataloged so far and *Lr1, Lr10, Lr21, Lr34*, and *Lr67* have been isolated and characterized through map based cloning approach (McIntosh et al., 2013; Bansal et al., 2017).

Rust fungi are obligatory biotrophic plant pathogens (Schulze-Lefert and Panstruga, 2003). *P. triticina* is a macrocyclic and heteroecious rust fungus with five spore stages and two host species (Bolton et al., 2008). The asexual uredinial stage on main host wheat is economically important stage and has potential to cause high losses under favorable weather conditions. Dikaryoticurediospores blown by wind or air are deposited on wheat leaf. Urediospores imbibe water, swell and develop a germ tube after coming into contact with a film of moisture. A germ tube is formed which continues to grow on leaf surface until a stoma is encountered. The germ tube forms an appressorium over the stomatal openings and the pore is entered by an infection peg (Hu and Rijkenberg, 1998). Six hours after inoculation, infection pegs develop substomatal vesicles (SSV). A primary infection hypha forms from SSV which attaches to a host cell. A septum forms cutting off the tip of the hypha delimiting a terminal haustorium mother cell (HMC) by 12 h. Host cell penetration begins with the formation of a penetration peg within an area of contact between HMC and host cell followed by formation of haustorium within the host cell. Haustoria are specialized hypha that act as feeding structures for the fungus (Bolton et al., 2008; Kolmer, 2013). After haustorial formation more infection hyphae are produced from HMC which also form HMCs and haustoria and result in a branching network of fungal mycelia. At 7–10 days post inoculation, mycelium gives rise to uredinia that produce dikaryotic urediniospres. Brownish urediniospores are released through ruptured epidermis. In highly susceptible hosts secondary uredinia form the primary pustules (Schafer, 1987).

The molecular analyses of host pathogen interactions have been restricted to limited number of genes (Casassola et al., 2015; Wang et al., 2015) but with the advances in sequencing technologies it has become affordable to study the complete transcriptomes of the host as well as pathogen. A number of studies have used ESTs (Manickavelu et al., 2010; Dmochowska-Boguta et al., 2015), microarrays (Fofana et al., 2007), serial analysis of gene expression (Chandra et al., 2016), and RNAseq (Zhang et al., 2014; Dobon et al., 2016) to understand the host pathogen relationship between known host genes and specific pathotypes under compatible as well as incompatible interactions.

The present study was planned to study the differential expression in a time course experiment using RNAseq of the whole transcriptome through comparative analysis in two genotypes differing for a leaf rust resistance gene *Lr57*. *Lr57* and a linked stripe rust resistance gene *Yr40* were introgressed from *Aegilops geniculata* chromosome 5M–5D of wheat (Kuraparthy et al., 2007). Linked leaf and stripe rust resistance genes are present on the telomeric end of short arm of chromosome 5D with introgression encompassing about 3.5% of 5DS. *Lr57* is an all stage resistance gene providing complete resistance to Indian, US and Australian isolates. The objectives of the present study were identification of differentially expressed genes in two genotypes, differing for the presence of *Lr57*, after challenge with pathogen followed by functional annotation and comparative analyses of the differentially expressed genes.

## Materials and methods

### Plant material and sample collection

A near isogenic line WL711+*Lr57* (T756; pau16062) containing leaf rust resistance gene *Lr57* introgressed from wild wheat species *A. geniculata* in wheat cv. WL711 background and the recipient parent WL711 were used for studying the differential expression (DE) under compatible and incompatible interactions with *P. triticina* pathotype 77-5. The near isogenic line was developed by crossing disomic substitution line DS5M (5D) in WL711 background with Chinese Spring stock having suppressor for *Ph1* locus for induction of homoeologous pairing. The F_1_ was again crossed and backcrossed with the recurrent parent WL711. Leaf and stripe rust resistant BC_3_F_1_ plants were selfed to generate BC_3_F_15_ introgression line which was used for transcriptome sequencing. The development of introgression lines carrying *Lr57* has also been described in Kuraparthy et al. (2007).

WL711 and WL711+*Lr57* were planted in sand:cocopeat mix in plastic trays in three replicates each and grown under 16:8 h light and dark cycle. First leaf of the 7-day-old seedlings of WL711 and WL711+*Lr57* were inoculated with *P. triticina* race 77-5 under screen house conditions in three biological replicates. Leaf samples were collected for total RNA extraction at five different time intervals (0, 12, 24, 48, and 72 h post inoculation; HPI) and stored in RNAlater® solution (Ambion) for further processing. One set of inoculated seedlings of WL711 and WL711+*Lr57* were grown for another fortnight to record disease development. Five time points for sampling were chosen based on the life cycle stages of the leaf rust pathogen (Hu and Rijkenberg, 1998). Initial time point selection of 0 HPI represent the contol mock inoculated leaves, while 12 and 24 HPI stages symbolized the development of haustorial mother cell and haustoria between mesophyll cells, respectively. The later two time scales (48 and 72 HPI) correspond to the further development in secondary hyphae, HMCs and huastoria leading to the development of networked fungal mycelia.

### RNA extraction, sequencing and assembly generation

Inoculated leaves from all three biological replicates from each time point were ground into a fine powder in liquid nitrogen by constant crushing using sterilized and chilled pestle and mortar to isolate RNA by using RNA Isolation kit (Qiagen). RNA quantity and quality was checked using Agilent Bioanalyzer. Libraries were constructed using TruSeq RNA library Prep Kit with insert size ranging from 266 to 297 bp and sequenced using high throughput sequencing through Illumina HiSeq2000. High quality paired end reads of 100 bp were generated. Reads were cleaned for adapter sequences and low quality bases were trimmed. The quality of reads was accessed using FASTQC toolkit (Anders and Huber, 2010). The adapter sequences were removed using cutadapter tool (Martin, 2011). *De novo* assembly of RNA-seq data was performed with Trinity software as no complete wheat genome data was available (Haas et al., 2013). The International Wheat Genome Sequencing Consortium (IWGSC) had released wheat draft genome sequence covering 64% of wheat genome which did not include the data from repetitive elements (IWGSC, 2014).

We BLAST searched the full length cDNA from Triticeae full-length CDS database (TriFLDB) with the IWGSC wheat genome with BLASTN at cut off *e*-value of 1e-5, which resulted in only partial hits. In the absence of suitable genomic reference, a reference transcriptome was generated by merging the high quality paired end reads of susceptible genotype WL711 at five different time intervals (0, 12, 24, 48, and 72 HPI), so as to cover the whole genome. The transcript contigs obtained were searched for *P. triticin*a sequences by performing standalone BLASTX with cut off *e*-value of 1e-3 against protein nr (NR) database, a total of 16,100 hits were found belonging to *Puccinia* and other microorganisms which were removed from further analysis.

### Measurement of gene expression levels and detection of differentially expressed genes

The normalized expression levels (FPKM: Fragment per kilobase of transcripts effective length per million fragments mapped to all transcripts) of each sample was calculated utilizing the uniquely mapped reads on the reference transcriptome. RSEM software was used to calculate the count values of transcripts for each sample at different time intervals utilizing the biological replicates (Li and Dewey, 2011). RSEM uses an iterative process to fractionally assign reads to each transcript based on the probability of the reads being derived from each transcript. To normalize the data among different libraries Trimmed mean of M-values (TMM) normalization was performed in Trinity. The WL711 RNA sequence at 0 HPI was considered as reference point for comparison and nine comparative datasets were generated which included identification of DEG at different time intervals w.r.t. mock inoculated control under compatible interaction in WL711 and incompatible interaction in WL711+*Lr57* at 12, 24, 48, and 72 HPI. The expected count values thus obtained were used with DESeq (Anders and Huber, 2010) and edgeR (Robinson et al., 2010) tools to identify the differentially expressed gene transcripts/isoforms (DEG) at false discovery rate (FDR) < 0.001. Transcripts showing at least 4-fold change in log2 values and false discovery-corrected statistical significance *P* < 0.001 were considered differentially expressed. Only those transcripts which were found differentially expressed by both DESeq and edgeR were used for further analysis. The DEGs in WL711 and WL711+*Lr57* were clustered based on the log transformed expression values by hierarchical clustering in R software.

### Functional annotation of differentially expressed genes and co-expression analysis

**Figure 1:**
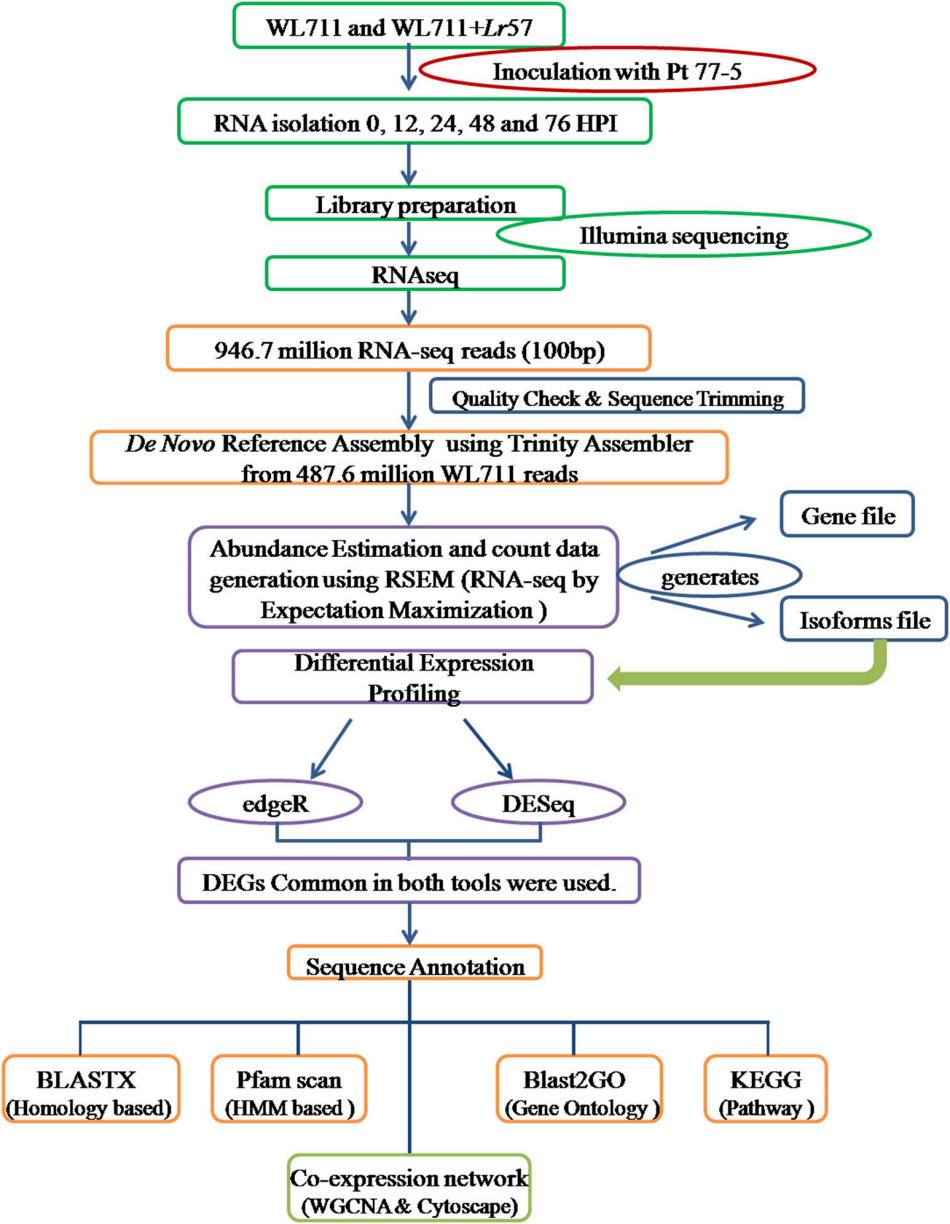
**Experimental set up for studying the differential gene expression in WL711 and WL711+Lr57 after challenge with *Puccinia triticina* pathotype 77-5**.

The identified transcripts gene/isoforms were annotated on the basis of corresponding homologs identified from blastx program against the NCBI protein “nr” database at *E*-value of 1e-3. Gene ontology terms and pathways associated with transcripts were determined using BLAST2GO program (Conesa and Götz, 2008). GO enrichment was analyzed within BLAST2GO using fisher's exact test with FDR value of 0.05. MapMan was used for functional analysis and visualization (Thimm et al., 2004). Protein families were assigned by searching against the Protein family (Pfam) database using HMM based tool pfamscan (Finn et al., 2014). The co-expression gene networks were developed in R using WGCNA package (Langfelder and Horvath, 2008). Log transformed FPKM values were used to identify the functional clusters/modules of highly co-expressed genes using adjacency matrix. The co-expression network were visualized in Cytoscape (Shannon et al., 2003). Separate modules were created for susceptible and resistant genotypes. The comparative GO terms in WL711 and WL711+*Lr*57 were plotted using wego (Ye et al., 2006). The experimental design is summarized in Figure 1.

## Results

### Generation of RNAseq data and *De novo* assembly

After stringent quality check and data cleaning 946.72 million high quality reads were obtained. For WL711 and WL711+*Lr57* a total of 174.5, 158.1, 155.0 and 149.00, 165.53, 144.59 million high quality reads, were obtained, respectively for each of the three biological replicates over five time points (Table 1). A reference transcriptome assembly was generated using *de novo* assembly program Trinity utilizing the 487.7 million reads belonging to 15 RNAseq datasets from WL711. The WL711 transcriptome assembly consisted of 376,398 transcript isoforms corresponding to 195,838 genes and was used as reference for the present study. Trinity groups transcripts onto clusters based on shared sequence content, these clustered are referred as gene and transcripts as isoforms. The N50 value evaluate the contiguity of the assembled sequences and is defined as the maximum length whereby at least 50% of the total assembled sequence resides in contigs of at least that length. An N50 of 1356 bp was achieved for the current transcriptome assembly. We identified a total of 16,100 transcripts belonging to *Puccinia* and other microorganisms, which were removed from the analysis. The related read data of five time interval of WL711 and WL711+*Lr*57 along with replicates (30 samples) have been deposited at National Center for Biotechnology Information (NCBI) in Short Read Archive (SRA) database under the accession number SRP078210. The assembled transcripts were deposited as Transcriptome Shotgun Assembly project at GenBank under the accession GEWU00000000.

**Table 1:** **Description of RNA-seq paired end data through Illumina-high throughput sequencing**.

**Genotype**	**Time interval (HPI)**	**Clean reads (Millions)**			**Total**
		**Replicate 1**	**Replicate 2**	**Replicate 3**	
WL711	0	40.39	27.46	25.91	93.76
	12	35.25	27.07	32.41	94.73
	24	34.61	27.67	29.87	92.15
	48	34.01	43.01	33.95	110.97
	72	30.28	32.90	32.92	96.10
Total reads		174.54	158.11	155.06	487.71
WL711+*Lr57*	0	36.10	27.59	31.25	94.94
	12	29.71	41.22	26.81	97.74
	24	25.20	32.14	25.12	82.46
	48	29.75	35.33	26.07	91.15
	72	28.24	29.25	35.34	92.83
Total reads		149.00	165.53	144.59	459.12

### Identification of differentially expressed genes

**Figure 2:**
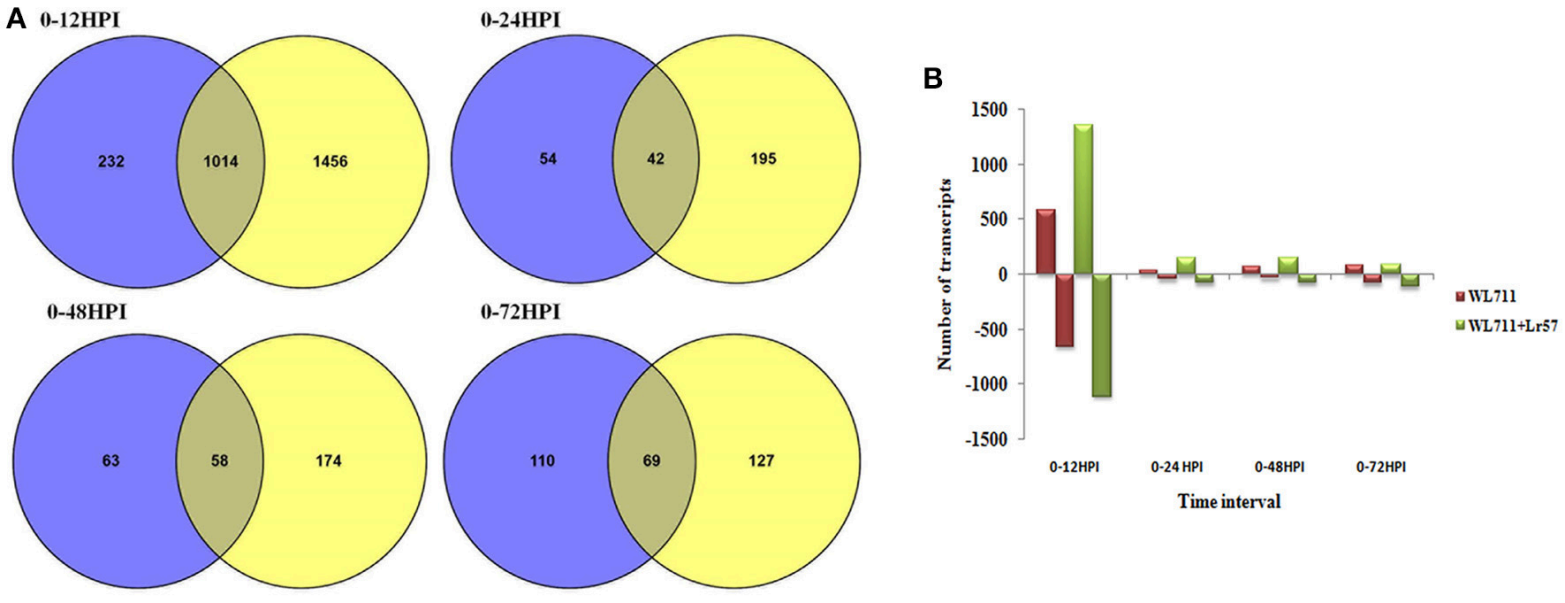
**(A)** Venn diagram showing overlap of differentially expressed genes between WL711 (blue) and WL711+*Lr57* (yellow) genotypes at different time points; **(B)** Regulation of differentially expressed genes between WL711 and WL711+*Lr57*.

A total of 1458 and 2692 transcripts were found to be differentially expressed in WL711 and WL711+*Lr57* over all the time points, respectively compared to the mock inoculated 0 HPI of the recipient genotype WL711 (Table 2). BLASTX homology search identified homologs for 2815 transcripts/genes most of which consisted of hypothetical, predicted and uncharacterized proteins while 121 and 186 genes remained un-annotated in WL711 and WL711+*Lr57*, respectively. A total of 1130 DE transcripts were common between both the genotypes including seven transcripts that belonged to genes encoding for proteins with nucleotide-binding and leucine rich repeats (NLRs) or resistance genes. Resistant genotype (WL711+*Lr57*) consisted of 59 distinct NLR encoding transcripts whereas susceptible genotype WL711 showed presence of only eight unique NLR encoding transcripts. The number of differentially expressed gene transcripts (DEG) between WL711 and WL711+*Lr57* at different time intervals is summarized in Table 2 and Figures 2A,B demonstrates the up- and down-regulated transcripts between WL711 and WL711+*Lr57*. Both the genotypes showed a peak of differentially expressed transcripts at 12 HPI when compared to other time points.

**Table 2:** **Differential Expression profiling in WL711 and WL711+*Lr57* at different time intervals**.

**Time Interval**	**RNAseq transcripts**	**No. of transcripts**					
		**Total**	**Unique**	**R genes**	**Up-regulated**	**Down-regulated**	**Unannotated**
0–12 HPI	WL711	1246	232	7	126	106	9
	WL711+*Lr57*	2470	1456	55	897	559	78
	Common^*^	1014	–	6	459	555	82
0–24 HPI	WL711	96	54	–	25	29	4
	WL711+*Lr57*	237	195	3	133	62	42
	Common	42	–	–	20	22	6
0–48 HPI	WL711	121	63	1	44	19	23
	WL711+*Lr57*	232	174	6	113	61	18
	common	58	–		37	21	22
0–72 HPI	WL711	179	110	2	63	47	26
	WL711+*Lr57*	196	127	1	57	70	18
	common	69	–	–	27	42	13
Overall	WL711	1458	328	8	–	–	121
	WL711+*Lr57*	2692	1562	59	–	–	186
	Common	1130	–	7	–	–	–

* *Expression of all the common transcripts were similar with slight variation in value. Overall in the time interval field represent the cumulative and distinct gene/transcript expression of WL711 and WL711+ Lr57*.

**Figure 3:**
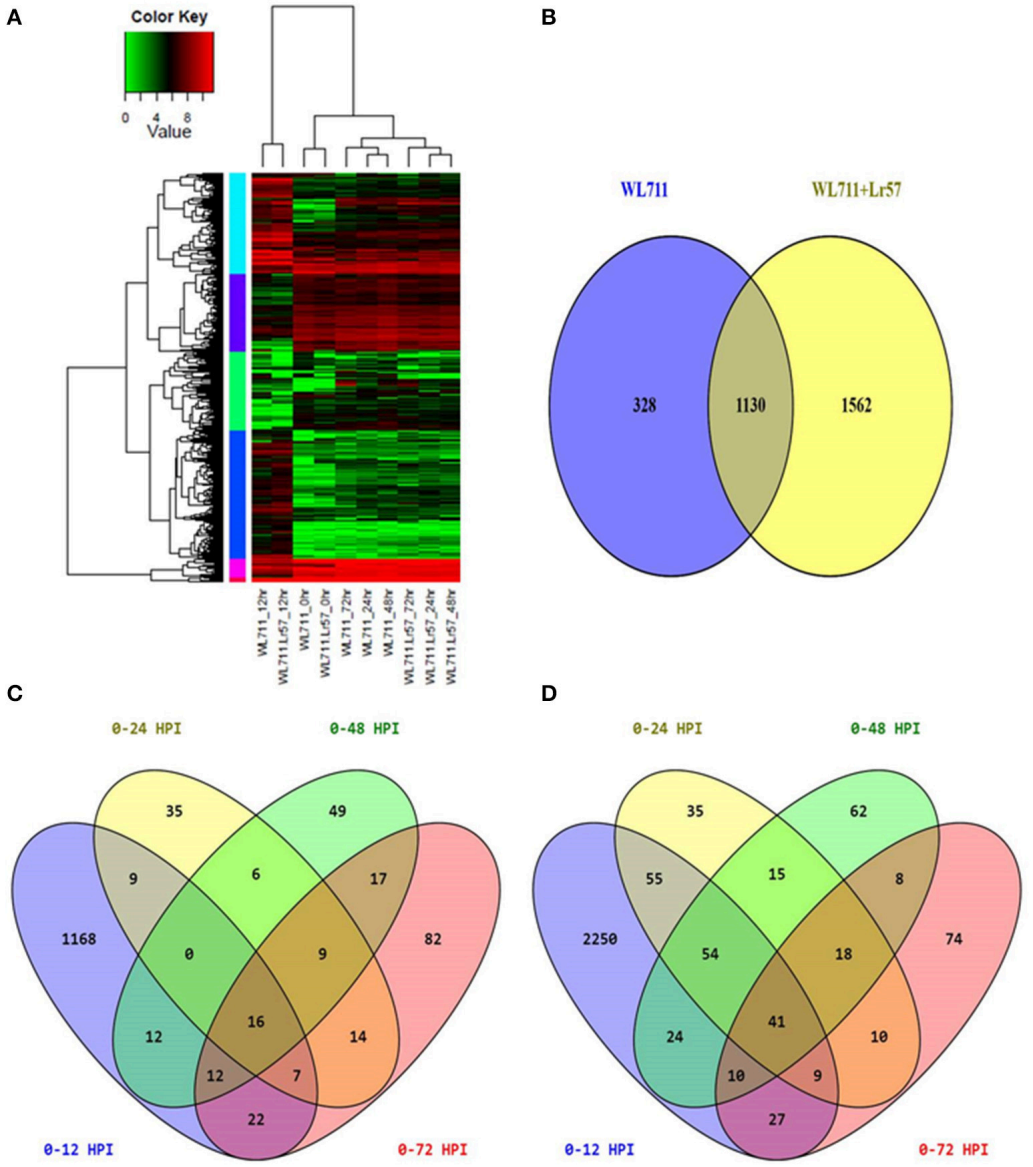
**(A)** Expression profiling of differentially expressed genes in both WL711 and WL711+*Lr57*. Horizontal row represents the gene and vertical columns denote samples. **(B)** Venn diagram showing the distribution of differentially expressed genes in WL711 and WL711+*Lr57*. **(C)** Distribution of differentially expressed genes at different time points post inoculation in WL711. **(D)** Distribution of differentially expressed genes at different time points post inoculation in WL711+*Lr57*.

Hierarchical clustering showed the presence of two major groups of DEGs. Group I consisted of differentially expressed transcripts in WL711 and WL711+*Lr57* at 12 HPI and Group II consisted of other eight data sets (Figure 3A). Transcripts from 0 HPI formed a sub-group within the second major cluster while 24, 48, and 72 HPI time points of WL711 and WL711+*Lr57* formed two distinct clusters may be owing to the large amount of distinct transcripts between two genotypes. Distribution of DEGs between WL711 and WL711+*Lr57* is depicted in Venn diagram (Figure 3B). The overlap between differentially expressed transcripts at different time points in WL711 and WL711+*Lr57* depicted that fewer genes were differentially expressed at later stages of infection (Table 2 and Figures 3C,D).

### Gene ontology based functional analysis of the DEGs

**Figure 4:**
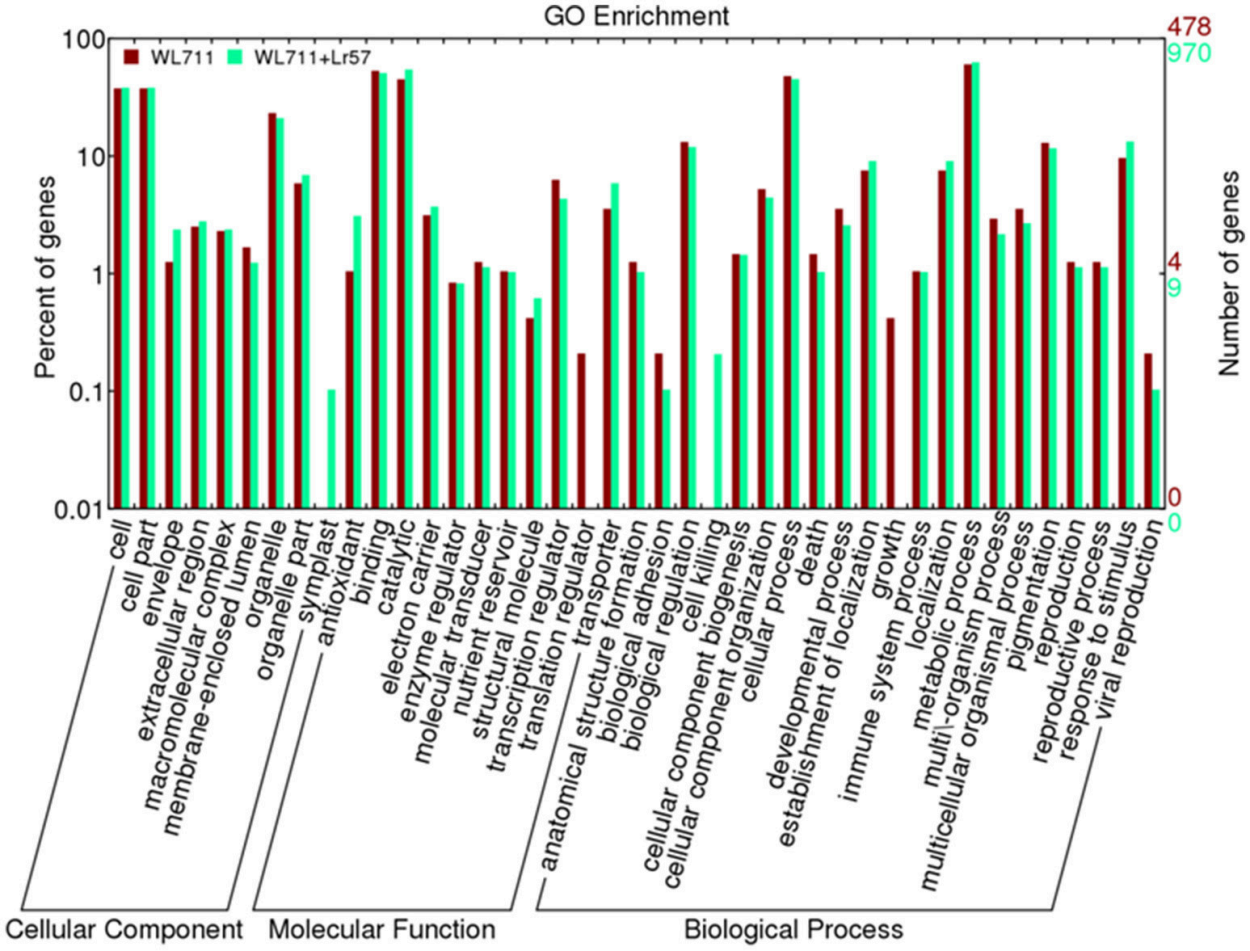
**Distribution of differentially expressed transcripts/genes involved in biological processes, molecular functions and cellular components in WL711 and WL711+*Lr57***.

Functional categorization of DEGs was done using Blast2GO. The gene ontology enrichment using the fisher's exact test at the false discovery rate of 0.05 depicts 26 and 22 over-represented gene ontology's in WL711 and WL711+*Lr*57, respectively (Table S1). Out of total 3020 DEGs in both the genotypes 988 transcripts were associated with at least one GO term. These were categorized into 41 functional groups in both genotypes (Figure 4). Biological process consisted of major portion of DEGs followed by molecular function and cellular component. In biological process metabolic process was most enriched with 288 and 609 DEG transcripts in susceptible and resistant genotype, respectively. Cellular process consisted of 299 transcripts of WL711 and 438 transcripts of WL711+*Lr57*. Response to stimulus and biological regulation were enriched more in resistant compared to susceptible genotype. GO term associated with immune system process consisted of 10 transcripts in WL711+*Lr57* and single transcript in WL711. In molecular function, catalytic activity (528) was the most abundant group followed by binding (493) in resistant genotype, while in susceptible genotype binding (254) was more prominent compared to catalytic activity (215). GO terms of transporter, transcription regulator, electron carrier, antioxidant, molecular transducer and nutrient reservoir activity consisted of 57, 42, 36, 30, 11, 10, and 17, 30, 15, 5, 6, 5 transcripts for resistant and susceptible genotypes, respectively. In cellular component category, most DEG transcripts belonged to cell in both genotypes, extracellular region was more enriched in resistant compared to susceptible genotype.

### Nucleotide-binding domain leucine rich repeat proteins

**Figure 5:**
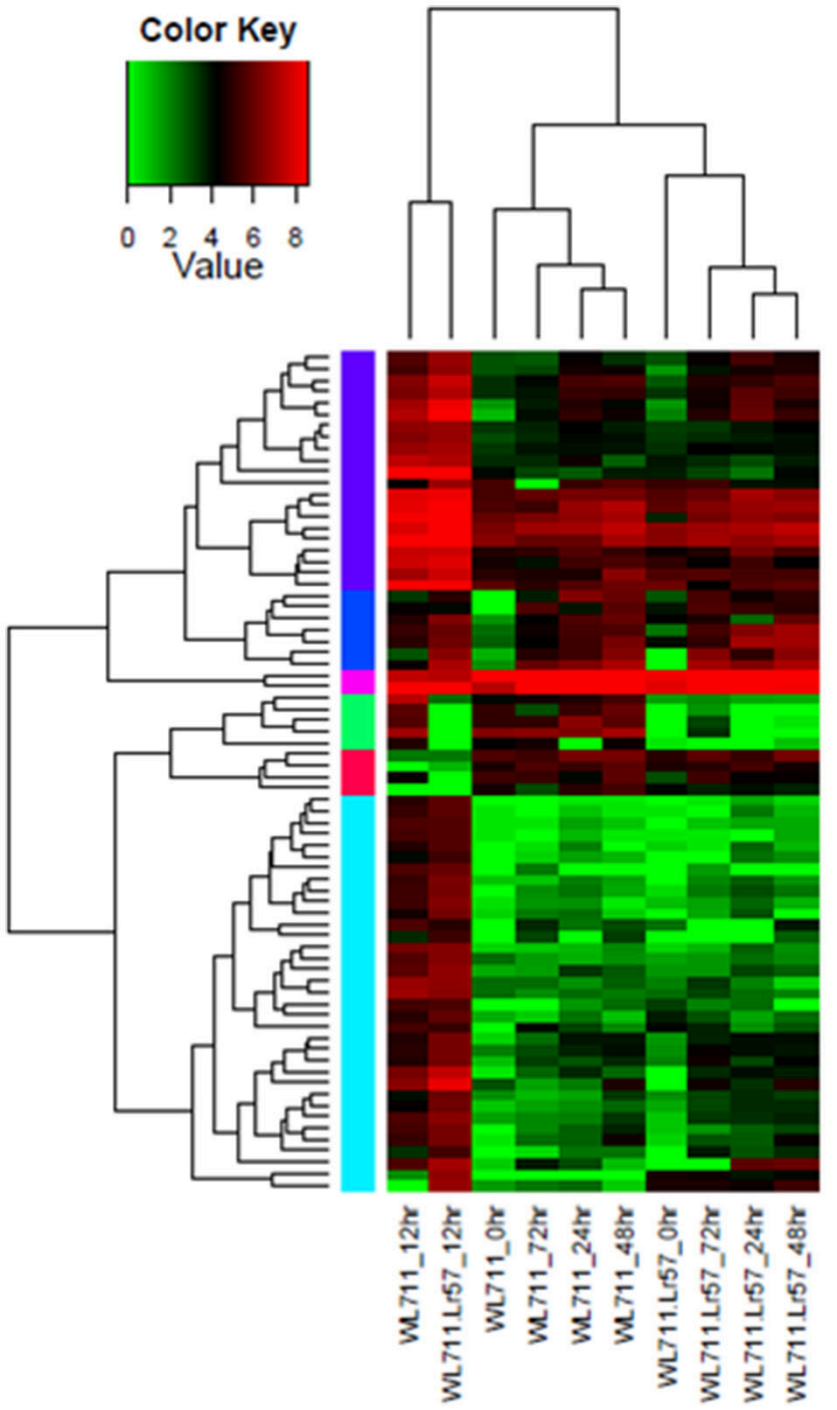
**Hierarchical cluster of 74 differentially expressed NLR genes in WL711 and WL711+Lr57 at different time points**. Hierarchical clustering of NLR genes showed peak induction of expression of resistance related genes immediately after infection with the pathogen i.e., at 12 HPI. Genes clusters are on the horizontal axis and samples on veritical axis. Colors on vertical represents the clustered genes based on gene expression, horizontal line represents the single gene and color of the line indicates the average gene expression in specific sample: high expression level in red, low expression level in green.

Fifteen NLR encoding transcripts showed differential expression in WL711 at different time points post inoculation while WL711+*Lr57* showed 66 differentially expressed NLR transcripts at different time intervals. Seven of these were common and showed similar expression behavior in both the genotypes within a range of 2- to 5-fold (Table S2). The expression of NLR encoding transcripts was found to be the most prominent around 12 HPI. Eight NLRs were found distinct to susceptible genotype with higher expression change at 12 HPI. Two NLRs with NB-ARC domain showed down-regulation at 12 and 72 HPI, respectively. Resistant genotype showed 59 distinct NLR encoding transcripts whose expression varied from 2.14- to 9.36-fold, out of these eight genes showed down-regulation during 0–12 HPI time point. Two NLR transcripts showed down-regulation upto 8-fold at 12, 24 and 48 HPI as compared to susceptible genotype. Three genes showed significant up-regulation at 48 HPI ranging from 4- to 5-fold, while one gene showed 8-fold up-regulation at 72 HPI only (Table S2). Distinct NLR genes in resistant genotype consisted of NB-ARC (25 gene), LRRNT_2 (22 gene), LRR_8 (1 gene), LRR_6 (1 gene), LRR_4 (10 gene) domains. The LRR_4 domain containing NLR transcripts showed variable expression during different stages of infection and NB-ARC domain containing genes showed large fold change in expression. Hierarchical clustering of NLR genes showed peak induction of expression of resistance related genes immediately after infection with the pathogen (Figure 5).

### Transcription factors related genes

**Figure 6:**
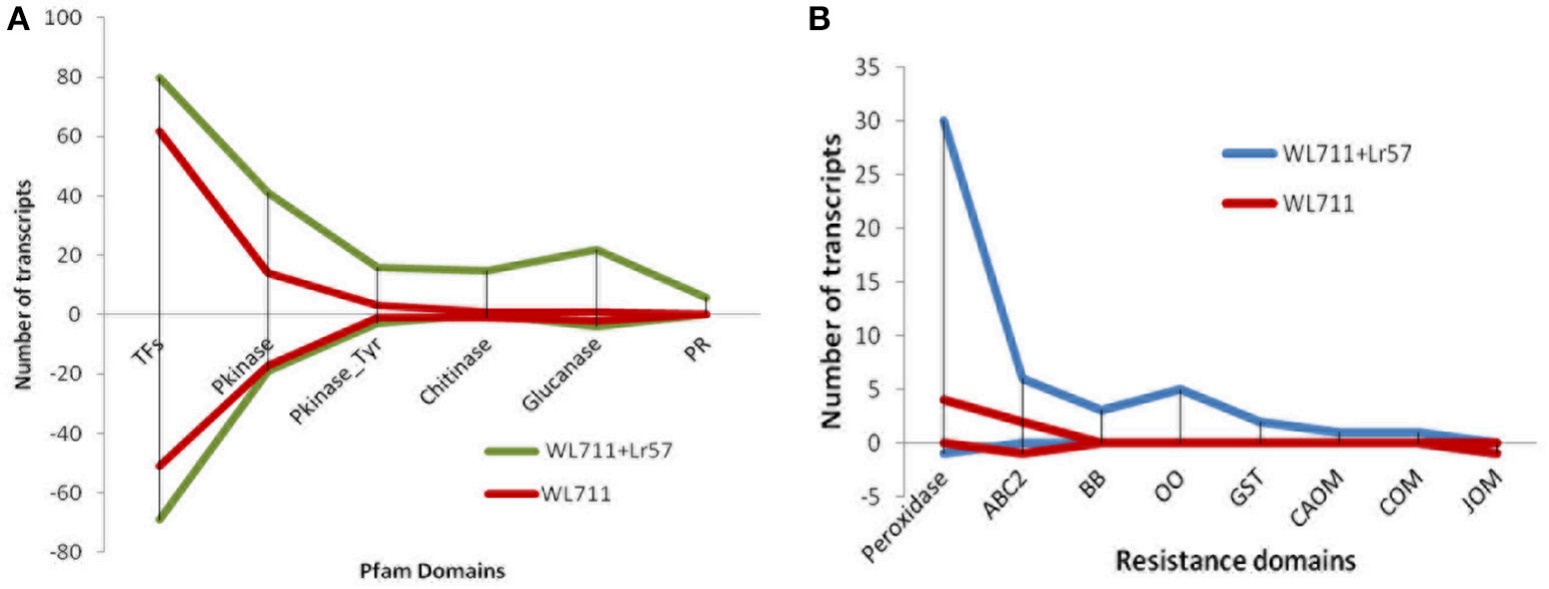
**Differential expression of genes (A)** Expression of genes for transcription factors, Pkinase, Pkinase_Tyr, Chitinase, Glucanase, and Pathogenesis-related (PR) proteins. **(B)** DEG for resistance gene domains for peroxidases, ABC2_membrane (ABC2), Bowman-Birk leg (BB), oxalate oxidase (OO), glutathione s-transferase (GST), caffeic acid 3-o-methyltransferase (CAOM), caffeoyl- o-methyltransferase (COM), and jasmonate o-methyltransferase (JOM) in WL711 and WL711+*Lr57*.

There were 118 and 159 DEGs belonging to transcription factors (TF) in susceptible and resistant genotypes, respectively. Of these 91 genes were common in both the genotypes. The susceptible genotype WL711 consisted of 27 TFs with the expression ranging from −9 to +9-fold. The resistant genotype consisted of 68 DE TFs with expression ranging from −10 to +8-fold. Forty eight TF genes were up-regulated in both the genotypes (Figure 6A). Twenty-two genes containing zinc finger domain were found to be down regulated in susceptible genotype (Table S3). Gene encoding NAM protein was found down-regulated to −2-fold at 12 and 72 HPI in resistant and only at 72 HPI in susceptible genotype. Three genes encoding zf-DOF domain were also found down-regulated to −2-fold in both the genotypes. Seven genes encoding MYB and IHY transcription factors were up-regulated to 2- to 4-fold in resistant genotype. Other TFs which played role in plant resistance like F-box, WRKY, B3, and Zip were found with the involvement of 13, 3, 1, 1 transcripts in WL711 and 14, 8, 2, 2 transcripts in WL711+*Lr57*, respectively.

### Genes related to protein kinase

Protein kinase play central role in signal recognition and activate the plant defense mechanism during the pathogen infection. In the present study 40 and 92 differentially expressed protein kinase genes were found in susceptible and resistant genotypes, respectively (Figure 6A). Twenty seven protein kinase with 15 down-regulated (−3.56 to −8.9-fold) and 12 up-regulated (2.13- to 10.88-fold) distinct transcripts were present in both genotypes. WL711 contained 13 unique protein kinase (seven down-regulated, five up-regulated) while WL711+*Lr57* demonstrate 65 distinct protein kinase, of which 12 showed down-regulation and 53 showed up-regulation (Table S4).

Among kinases, cysteine-rich receptor-like protein kinase forms the major class (six genes, 2- to 9.24-fold) which was expressed only in resistant genotype at 12 HPI. Three genes related to wall-associated receptor kinase were identified; their expression varied from 2.44- to 4-fold, one gene encoding calcium-dependent calmodulin-independent protein kinase was up-regulated with 2-fold change in expression. Eleven genes with lectin-domain containing receptor kinase were found to be expressed at 12 HPI and one of them tid_101283_c0_seq2 showed expression at 24 and 48 HPI (Table S4).

### Chitinase and glucanase genes

Chitinases and glucanases play an important role in plant defenses against the fungal pathogens. WL711 showed presence of only two chitinase genes belonging to GH19 family one of which was down regulated and other was highly up-regulated upto 7-fold (Figure 6A). Whereas WL711+*Lr57* demonstrated 16 genes belonging to GH19 and GH18 family, out of which four genes showed continued up-regulation upto 48 HPI. Five glucanase genes were identified in WL711 belonging to GH17 and GH16 family of which two were up-regulated while WL711+*Lr57* showed 26 DEG of which 22 were up-regulated while four were found down-regulated. These were the same genes which were down-regulated in WL711 (Table S5).

### Pathogenesis-related protein

Pathogenesis-related (PR) proteins are produced by the plant in response to a pathogen attack. Nine gene transcripts were found belonging to pathogenesis related protein in resistant genotype WL711+*Lr57*, all of which showed up-regulation and five of which were differentially expressed up to 48HPI (Figure 6A and Table S5). No PR-proteins were identified differentially expressed in susceptible genotype. Protein domain AvrRpt-cleavage is a conserved domain found in RIN4 protein (resistance R membrane–bound host-target protein) which is a target for typeIII virulence effector AvrRpt2 and Avrb. We identified single gene corresponding to AvrRpt-cleavage domain, which showed down-regulation to 8-fold at all stages in susceptible plant while it was down-regulated to 8-fold at 72 HPI in resistant genotype but it was not differentially expressed at other stages though it might be having the basal expression.

### Domains involved in plant resistance

In WL711+*Lr57 three* transcripts corresponded to bowman-birk type trypsin inhibitor were identified at 0–12 HPI with 4.4- to 8.6-fold expression whereas in WL711 no bowman-birk inhibitor was found (Table S6 and Figure 6B). ABC2_membrane domain, also known as transporters were DE in both the genotypes. In WL711 two transcripts were expressed at 12 HPI with expression ranging from 3.09- to 11.10-fold (Figure 6B), one gene encoding ABC transporter g family member 14 showed down regulation at 12 and 48 HPI. While WL711+*Lr57* consisted of seven genes, six of which shows 5- to 10-fold increase in expression and one gene showing down regulation at 24 HPI while in WL711 same was downregulated at 12 and 48 HPI. One gene of oxalate oxidase 2 and four isoforms of gene oxalate oxidase were up-regulated with expression ranging from 3.2- to 8.5-fold in resistant genotype at 12 HPI (Figure 6B). Three transcripts related to caffeic acid 3-o-methyltransferase (plays important role in the lignin biosynthetic pathway) were found of which one was up-regulated at 12 HPI and two showed up-regulation at 72 HPI. Caffeoyl-o-methyltransferase and jasmonate o-methyltransferase were also differentially expressed at 0–12 HPI in WL711+*Lr57* where their expression was 2.9- and 5.5-fold, respectively (Figure 6B). While in susceptible genotype only transcript belonging to jasmonate o-methyltransferase was found downregulated upto −8.5-fold. Phenylalanine ammonia-lyase (PAL) showed expression of 3.2- to 8.4-fold in resistant genotype at 0–12 HPI with the involvement of six transcripts whereas in susceptible plant only one transcript was expressed having expression of 4.8-fold. Plant glutathione S-transferase was up-regulated with two genes in resistant plant at 12 HPI with 3.6- and 4.2-fold change. Resistant genotype consisted of 31 transcripts belonging to peroxidases which showed an indication of increased oxidative burst at the site of infection (Figure 6B). Two transcripts (tid_119230_c1_seq4, tid_118794_c0_seq7) showed continued expression till 24 HPI. Thirty one and thirty eight transcripts belonging to Chlorophyll a-b binding protein were down- regulated in WL711 and WL711+*Lr57*, respectively at all time intervals. These differentially regulated plant resistant domains has been specified in Table S6.

### Pathways induced during *Puccina triticina* infection

**Figure 7:**
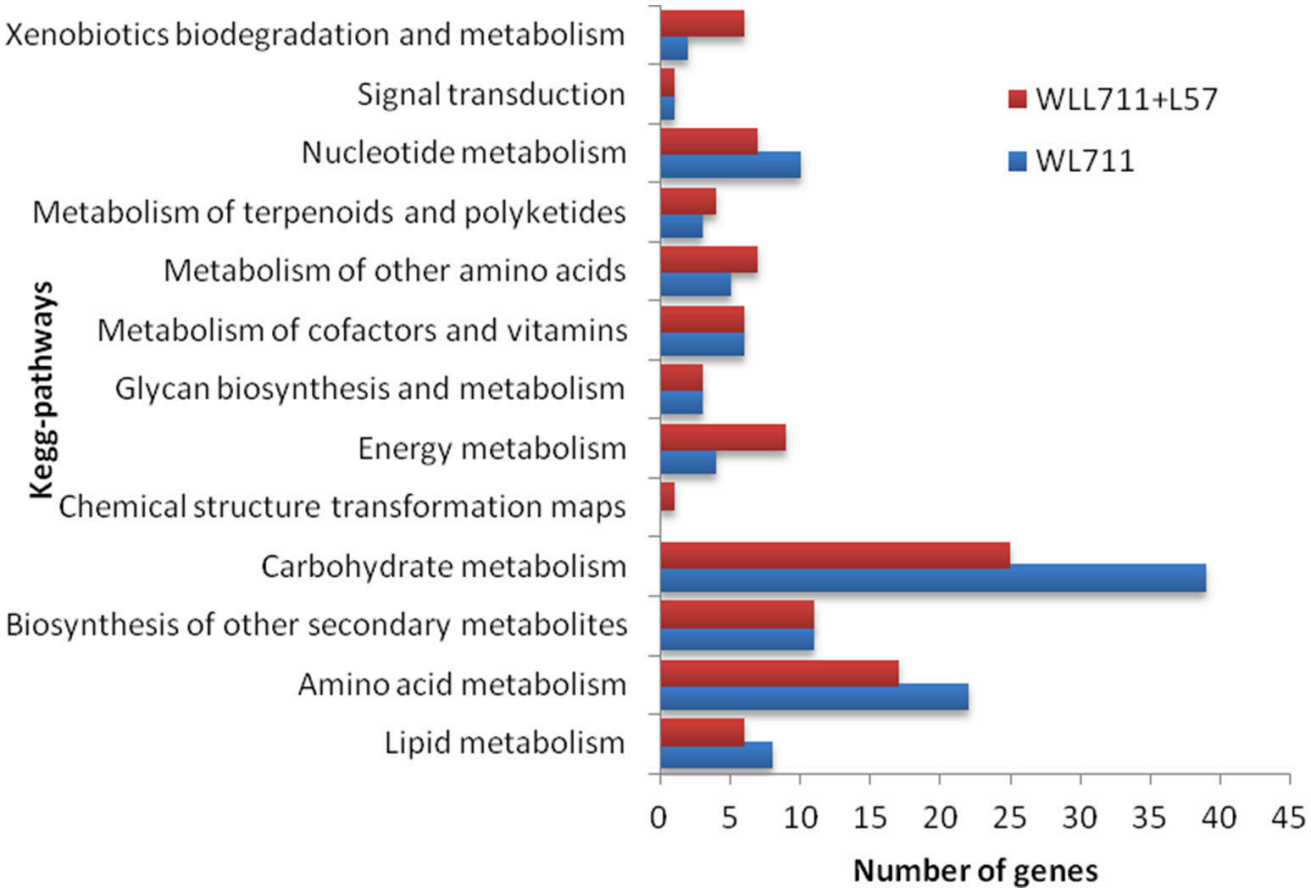
**Number of differentially expressed pathways between susceptible and resistant genotypes WL711 and WL711+*Lr57*, respectively**.

We mapped the DEG transcripts of WL711 and WL711+*Lr57* to KEGG database to identify significant pathways. DEG transcripts with KEGG annotation were categorized belonging to 42 and 59 different pathways in WL711 and WL711+*Lr57*, respectively, of which 39 were common between the two genotypes. We manually classified these pathways into 13 major KEGG classes (Figure 7). In both the genotypes carbohydrate metabolism was the most prominent pathway followed by amino acid metabolism; biosynthesis of other secondary metabolites and nucleotide metabolism. Pathways of glycan biosynthesis and metabolism; metabolism of cofactors and vitamins; metabolism of terpenoids and polyketides were found with similar number of DEGs (Table 3). In WL711, starch and sucrose metabolism consisted of maximum number of 17 DEG transcripts while in WL711+*Lr57*, phenylalanine metabolism which participated in plant defense was expressed most with the involvement of 35 DEG transcripts. The secondary metabolite pathways playing role in plant defense were expressed in both the genotypes. In WL711, isoquinoline alkaloid biosynthesis; phenylpropanoid biosynthesis; terpenoid backbone biosynthesis; tropane, piperidine, and pyridine alkaloid biosynthesis showed differential expression with three genes each and indole alkaloid biosynthesis; streptomycin biosynthesis were DE with one gene each. In WL711+*Lr57*, Phenylpropanoid biosynthesis was most differentially expressed with involvement of 33 genes/transcripts making it the most prominent secondary metabolism pathway followed by terpenoid backbone biosynthesis with five genes while others showed expression similar to susceptible genotype. Some pathways which help the plant to fight against various types of stresses such as phenylalanine metabolism; thiamine metabolism; phenylalanine, tyrosine and tryptophan biosynthesis; glutathione metabolism; riboflavin metabolism showed differential expression. Pathways of alanine, aspartate and glutamate metabolism; drug metabolism; taurine and hypotaurine metabolism; biosynthesis of terpenoids and steroids; flavonoid biosynthesis; metabolism of xenobiotics by cytochrome P450; tryptophan metabolism were DE in resistant genotype (Table 3).

**Table 3:** **Important pathways identified in resistant and susceptible genotypes**.

**Pathway**	**Number of transcripts/genes**		
	**Enzymes**	**WL711+*Lr57***	**WL711**
Phenylalanine metabolism	Oxidase	3	2
	Ammonia-Lyase	6	−
	Lactoperoxidase	25	3
	O-Methyltransferase	1	−
Phenylpropanoid biosynthesis	Ammonia-Lyase	6	−
	Lactoperoxidase	25	3
	O-Methyltransferase	1	−
	Dehydrogenase	1	−
Starch and sucrose metabolism	Alpha-Glucosidase	11	6
	Endo-1,3-Beta-D-Glucosidase	1	−
	Saccharogen Amylase	3	3
	Invertase	11	6
	Synthase	2	2
	Trehalose 6-Phosphatase	4	−
Galactose metabolism	Phosphohexokinase	1	−
	1-Epimerase	7	2
	Invertase	11	6
Amino sugar and nucleotide sugar metabolism	4-Epimerase	5	2
	Chitodextrinase	10	1
	Transaminase (Isomerizing)	1	−
Purine metabolism	Phosphatase	9	2
	RNA Polymerase	3	3
	Adenylpyrophosphatase	3	1
Thiamine metabolism	Phosphatase	9	2
	Synthase	3	3
Beta-Alanine metabolism	Oxidase	3	3
	Decarboxylase	4	−
Glycerophospholipid metabolism	Kinase	2	2
	Phosphodiesterase	2	2
	Cytidylyltransferase	3	−
	Phosphatase		1
Glyoxylate and dicarboxylate metabolism	Equilase	1	−
	Phosphatase	3	3
	Carboxylase	2	1
Pyrimidine metabolism	RNA Polymerase	3	3
	Reductase	1	1
	Phosphoribosyl Transferase	1	−
	Decarboxylase	1	−
Pyruvate metabolism	Dehydrogenase (Oxaloacetate-Decarboxylating)	4	2
	Lyase	1	−
Arginine and proline metabolism	5-Kinase	1	−
	Decarboxylase	3	3
	Kinase	1	−
Cysteine and methionine metabolism	O-Acetyltransferase	1	−
	Synthase	1	−
	Decarboxylase	3	3
Glycine, serine and threonine metabolism	Oxidase	3	2
	Kinase	2	−
Terpenoid backbone biosynthesis	Reductoisomerase	1	−
	Synthase	3	3
	2,4-Cyclodiphosphate Synthase	1	−
Riboflavin metabolism	Phosphatase	3	3
Isoquinoline alkaloid biosynthesis	Oxidase	3	3
Phenylalanine, tyrosine and tryptophan biosynthesis	Dehydratase	1	1
	Dehydrogenase	2	2
Tropane, piperidine and pyridine alkaloid biosynthesis	Oxidase	3	3
Drug metabolism - cytochrome P450	Monooxygenase	1	1
	Transferase	1	
Glutathione metabolism	Dehydrogenase (NADP+)	1	1
	Transferase	1	
Indole alkaloid biosynthesis	Synthase	1	1
Phosphatidylinositol signaling system	Phosphatase	1	1
Streptomycin biosynthesis	Phosphatase	1	1
Alanine, aspartate and glutamate metabolism	Decarboxylase	4	−
	Transaminase (Isomerizing)	1	−
Drug metabolism - other enzymes	Ali-Esterase	3	−
	Phosphoribosyltransferase	1	−
Taurine and hypotaurine metabolism	Decarboxylase	4	−
Biosynthesis of terpenoids and steroids	Synthase	1	−
Flavonoid biosynthesis	O-Methyltransferase	1	−
Metabolism of xenobiotics by cytochrome P450	Transferase	1	−
Tryptophan metabolism	Equilase	1	−

### Gene co-expression network analysis

**Figure 8:**
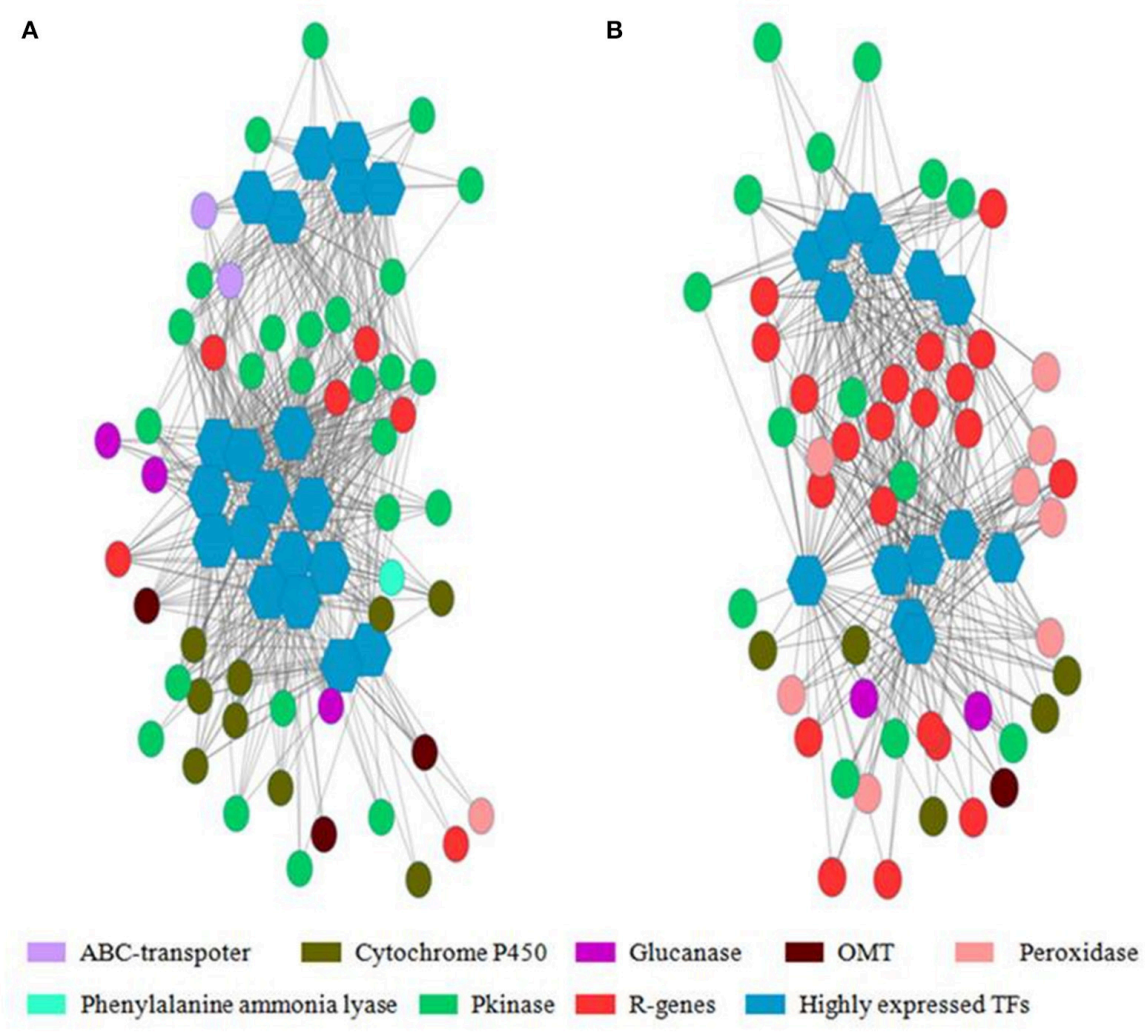
**Co-regulated genes expression network of highly expressed transcription factor with other resistance genes**. Hexagon represents the transcription factors with more than 20 connections with nearby genes and circle represents the resistance gene, peroxidases, glucanases, cytochrome P450, ABC transporters, Phenylalanine ammonia lyase, protein kinases and O-methyltransferase. Edges represents the degree of connectivity in depicted by weighted gene matrix. **(A)** Gene network from cluster 1 of WL711. **(B)** Gene network from cluster 1 of WL711+*Lr*57.

Weighted Gene Co-Expression Network Analysis (WGCNA) was used for finding gene clusters/modules of co-expressed genes (Figure S1). WGCNA is an R software package which performs weighted correlation network based on adjacency matrix developed using gene expression data. In the network gene/transcript were represented as node and the weighted edges as connection between the nodes. Using WGCNA, 4 modules (cluster of highly correlated genes) were identified in WL711 where NLR encoding transcripts were mostly present in module 1 (13 NLR genes). Transcription factors were present in all the modules. For the resistant genotype 8 modules were produced and NLR genes were present in 4 modules. Cluster one was rich in NLR genes with count of 50 genes. Other three modules were having 4, 2, and 3 NLR genes, respectively. All the modules except 8 had transcription factors. (Figure 8 and Table S6). The gene co-regulation network of highly expressed transcription factors is shown in Figure 8 depicting the strong relationship of resistance gene (R-genes) with protein kinases and peroxidases in Cytoscape network.

### Differential expression profiles of NLR genes in susceptible and resistant genotypes at different time points and identification of potential candidate genes

Fifteen NLR encoding transcripts showed differential expression in WL711 while WL711+*Lr57* showed 45 genes with 57 differentially expressed isoforms at different time points. Three of these were common in both and showed similar expression behavior at 0–12 HPI in both the genotypes within a range of +2.1 to +5.7-fold. Amongst the 12 unique NLR genes of susceptible genotype, a total of 10 genes were differentially expressed at 12 HPI with 9 genes being up-regulated with expression range of +2.2 to +8.3-fold and one being down-regulated to −8.0-fold (Table 4).

**Table 4:** **Summary of the differentially expressed NLR genes in WL711 and WL711+*Lr57***.

NLR-genes^*^	**WL711**		**WL711+*****Lr57***	
	**Number of transcripts**	**Expression range**	**Number of transcripts**	**Expression range**
LRR_1	1	+2.2	0	0
LRR_4	3	+2.7 to +8.6	16	+2.6 to +10.8
LRR_6	3	+2.7 to +4.2	1	+2.1
LRRNT_2	2	−8.5 to +3.9	23	+2.5 to +10.1
NB-ARC	6	−8 to +8.3	17	−9.6 to +8.9

* *Protein family domains:Leucine Rich Repeat family: LRR_1(PF00560), LRR_4(PF12799), LRR_6(PF13516); Leucine rich repeat N-terminal domain:LRRNT_2 (PF08263); NB-ARC (PF00931)*.

The resistant genotype expressed 54 unique R-genes and 42 were DE only at 12 HPI, amongst these 39 genes were up-regulated with +2.1 to +10.8-fold variation and moreover, the three down-regulated transcripts encoding for NB-ARC domain showed expression of −4.0 to −9.6-folds (Table 4). Distinct NLR-genes in the resistant genotype consisted of LRRNT_2 (23 genes with up-regulation), NB-ARC (17 genes with 4 being down-regulated), LRR_4 (16 genes with up-regulation). LRR_4 domain containing genes showed variable expression during different stages of infection (Table 4).

To understand the change in expression at transcript level in response to the infection we compared the expression between the different time intervals utilizing the expected count values of each resistant gene identified through RSEM tool. In WL711 eight transcripts/genes were having the basal expression which increased upto 2- to ~5-fold at 12 HPI. One gene having the basal expression was down-regulated at 12 HPI showing a consistent expression at later stages but did not qualify as differentially expressed. Four transcripts showed zero basal expression which increased to 8-fold at 12 HPI. So an induced effect can be seen on pathogen invasion.

In WL711+*Lr57*, 20 transcripts belonging to 17 genes showed the basal expression. Sixteen transcripts showed 2- to 5-fold change in expression. Thirty six transcripts belonging to 29 genes were having zero basal expression which increased to 4- to 10-fold at 12 HPI. Two genes (tid_120436_c1_seq6; tid_108334_c0_seq7) showed the continuous differential expression of 8- to 9-fold upto 48 HPI.

The putative candidate defense related genes were identified using the absolute value of FPKM ratio >100 for the WL711+*Lr57* at 12 HPI, which resulted in nine genes with 5- to 6-fold change in expression. One of the genes we selected (tid_120436_c1_seq6) was having the zero basal expression but the expression changed to +9-fold at later stages (Table 5). When selected, the transcripts with >500 FPKM ratio, seventeen new genes were identified belonging to transcription factor, peroxidases, chitinases (Table 5). Among these the two glycol_hydro_17 genes and one DIOX_N domain containing gene showed continued increased expression till 48 HPI. Two genes (tid_48246_c0_seq1, tid_48396_c0_seq1) for which we have no annotation showed expression of +8 to +9-fold continuous expression.

**Table 5:** **Transcripts/genes present exclusively/highly expressed in the resistant genotype WL711+*Lr57***.

**Transcripts**	**FPKM ratio**				**Pfam domain**	**Fold-change**			
	**0–12 HPI**	**0–24 HPI**	**0–48 HPI**	**0–72 HPI**		**0–12 HPI**	**0–24 HPI**	**0–48 HPI**	**0–72 HPI**
tid_102183_c0_seq1	152.67	9.67	20.33	12.00	LRRNT_2	6.24	−	−	−
tid_108238_c0_seq15	119.60	10.20	29.60	10.20	NB−ARC	5.83	−	−	−
tid_110918_c0_seq1	120.07	13.07	6.79	6.43	LRRNT_2	5.73	−	−	−
tid_110918_c0_seq5	125.54	21.32	11.64	9.42	LRRNT_2	6.00	−	−	−
tid_110918_c1_seq1	129.60	25.47	10.84	9.49	LRRNT_2	6.20	−	−	−
tid_120436_c1_seq6	infinity	infinity	infinity	infinity	LRR_4	9.75	9.34	9.87	−
tid_124955_c0_seq7	121.53	8.82	3.94	1.94	LRR_4	5.99	−	−	−
tid_46507_c0_seq1	104.50	12.92	14.33	10.67	NB−ARC	5.60	−	−	−
tid_56324_c0_seq2	143.67	11.33	3.50	0.00	LRRNT_2	6.12	−	−	−
tid_95578_c0_seq1	144.17	0.00	4.83	1.00	LRRNT_2	6.03	−	−	−
tid_107549_c0_seq1	641.29	18.35	5.08	7.46	Peroxidase	8.22	−	−	−
tid_117723_c1_seq6	553.06	1.32	0.87	2.89	DUF1685	8.06	−	−	−
tid_91820_c0_seq1	1334.33	132.67	76.17	71.50	DUF674	8.96	−	−	−
tid_101041_c0_seq1	688.00	1.82	0.00	5.00	Cupin_1	8.58	−	−	−
tid_120482_c0_seq8	1096.14	2.45	0.31	0.14	Myb_DNA−binding	9.19	−	−	−
tid_48246_c0_seq1	1185.17	1509.67	521.67	987.83	−	8.96	9.59	8.07	9.03
tid_48396_c0_seq1	1024.13	1168.88	392.00	768.12	−	9.12	9.57	8.01	9.02
tid_108852_c0_seq1	625.00	199.00	514.00	224.00	Pkinase	8.32	−	−	−
tid_109512_c1_seq1	1448.75	72.50	14.63	17.63	peroxidase	9.46	−	−	−
tid_110930_c0_seq4	683.95	102.09	100.86	206.26	Glyco_hydro_17	8.28	5.93	5.87	−
tid_114760_c3_seq1	537.50	73.11	67.81	148.56	Glyco_hydro_17	7.87	5.39	5.24	−
tid_114849_c1_seq30	626.00	123.50	5.50	119.50	PP2C	8.32	−	−	−
tid_115376_c1_seq18	1964.00	593.00	248.00	135.00	WRKY	9.27	−	−	−
tid_116007_c0_seq5	3980.00	1585.00	1493.00	4126.00	DIOX_N	10.52	9.19	9.13	−
tid_120107_c2_seq18	751.00	392.00	394.00	399.00	−	8.40	−	−	−
tid_120107_c2_seq22	855.00	234.50	341.00	479.00	−	9.53	−	−	−
tid_82828_c0_seq1	864.47	72.17	73.63	224.30	−	8.33	−	−	−

We blast search the differentially expressed genes against the wheat genome at ensemble plants. In WL711+*Lr57* three R-genes having LRRNT domain were mapped to chromosome 5D of wheat. Transcripts tid123169_c0_seq2 and tid125064_c2_seq1 showed 96 and 97% identity to gene sequence in database, both were having 2-fold increase in expression (Table S2) and were mapped to the short arm of chromosome at same location. These two transcripts showed 98% identity. Further investigation shows tid125064_c2_seq1 is a homoeologous sequences from chromosome 5B. Transcript tid124758_c1_seq4 mapped to long arm of 5D with 98% identity. These transcripts were missed in FPKM ratio due to their lower expression change. We further mapped the raw reads of seventy four NLRs transcripts. The reads which mapped potential candidate gene tid123169_c0_seq2 from WL711+*Lr57* were extracted and assembled into a single contig using spade assembler. The assembled transcript was 2767 bp as compared to 2761 of WL711 (Figure S2). The sequence alignment in clustalx showed an indel of 6 bp and 42 single nucleotide differences reflected in 15 amino acids betweenWL711 and WL711+*Lr57* The genes consist of active protein kinase domain along with the leucine rich repeats, which conforms their role in signal transduction.

## Discussion

Plants defend themselves against pathogenic microbes or pests by relying on innate immunity and lack specialized immune system (Macho and Zipfel, 2014). Surface pattern recognition receptors (PRRs) present the first level of defense that recognize pathogen-associated molecular pattern (PAMPs) to induce basal resistance response called pattern triggered immunity or PTI. PTI is an important barrier against disease and prevents non-adapted microbes from infecting (Cui et al., 2015). Adapted pathogens have evolved virulence factors called effectors which are delivered inside the host cells to interfere with PTI. This drives host-pathogen coevolution and plants use a family of polymorphic intramolecular nucleotide-binding/leucine-rich-repeat (NLR) receptors that recognize pathogen effectors and induce a strong resistance response called effector triggered immunity or ETI. ETI is often associated with local plant cell death also referred to as hypersensitive response. *Lr57* leaf rust resistance gene investigated in the present study also induced a hypersensitive response and is effective throughout the plant life. Most of the disease resistance genes cloned in wheat so far, are NBS-LRRs. Two differentially expressed transcripts were BLAST search to wheat genome and mapped on short arm of wheat chromosome 5DS, the location where *Lr57* has been mapped by Kuraparthy et al. (2007). In addition to this we also identified differential NLRs in the compatible and incompatible interactions with 54 unique NLRs in resistant genotype. Seventeen of these genes showed basal expression indicating that these might be expressed throughout the plant life while 29 genes were induced upon infection at 12 HPI indicating that these genes might be involved in ETI. Two of these genes showed upregulation even upto 48 HPI. A peak of NLR gene expression was seen at 48 HPI in compatible and incompatible interaction by Dobon et al. (2016) with drop in expression in stripe rust susceptible host at 72 HPI and continued upregulation in resistant host. We observed a sharp increase in gene activity in both genotypes at 12 HPI but the number of the NLR genes was higher in resistant genotype and expression of the NLR genes in resistant genotype continued to increase upto 48 HPI. Active suppression or alteration of upstream signaling by the leaf rust likely resulted in suppression of immune response and proliferation of the pathogen in compatible interaction.

**Figure 9:**
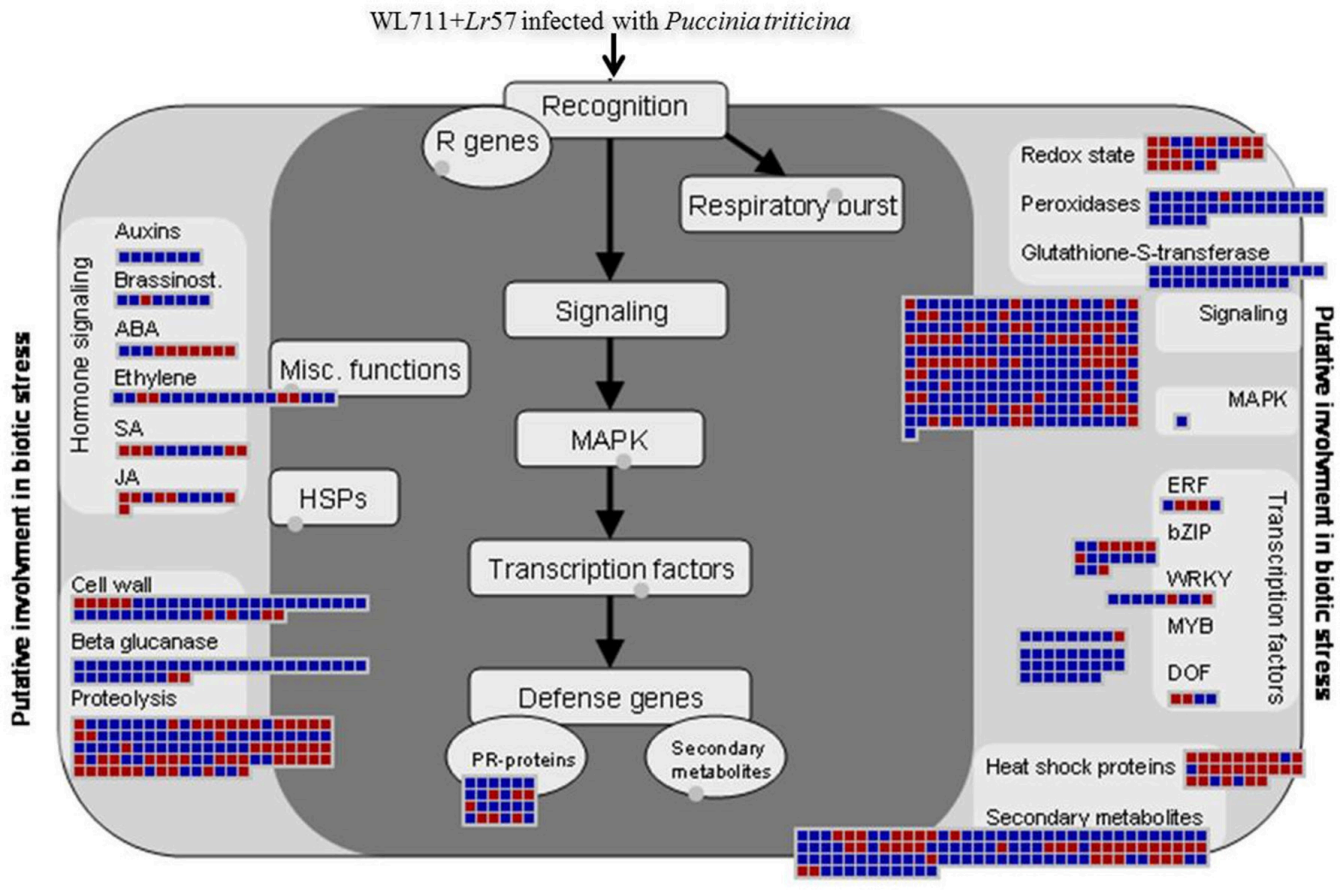
**MapMan visualization of defense response in WL711+*Lr*57 after pathogen attack. DEGs (fold changes ≥ 2, *p* ≤ 0.05) were imported into MapMan**. Up- and Down-regulated DEGs are represented with blue and red squares, respectively with log2 transformed values.

Aburst of gene activity of different GO categories besides NLR genes was observed upon infection in both susceptible and resistant genotype with incompatible interaction more enriched in defense related genes. This complexity in host-pathogen interactions can be understood as the process which involves cascade of signaling processes consisting of different transcription factors hormones, secondary metabolites, signaling molecules (receptors kinases), peroxidases, PR proteins, etc. Figure 9 summarizes different categories and differential expression patterns in WL711+*Lr*57.

A global gene expression approach is useful for elucidating the molecular mechanisms of wheat–fungus interactions, particularly the application of next-generation sequencing to study important non-model host–pathogen systems, such as wheat rusts (Cantu et al., 2013). Some studies have studied wheat–rust interactions to identify/locate the key genes involved in resistance mechanism such as those using the Affymetrix® GeneChip® Wheat Genome Array (Hulbert et al., 2007; Coram et al., 2009), EST sequencing (Manickavelu et al., 2010), cDNA-AFLP analysis (Bruce et al., 2014), SAGE (Chandra et al., 2016), and RNAseq (Zhang et al., 2014; Dobon et al., 2016).

A sudden burst of differential gene expression was observed at initial stages after infection i.e., 12 HPI assuming plant mounts all its defense responses immediately after encounter with the pathogen. This scenario was apparent in both resistant and susceptible genotypes. Differentially expressed NLR transcripts were more abundant in WL711+*Lr57* than in WL711. Transcription factors play an important role in controlling the gene expression. We observed an abrupt change in expression of transcription factors after inoculation in both the genotypes. At least 48 TFs were upregulated in both the genotypes while MYB and IHY transcription factors were specifically up-regulated in resistant genotype. Signal transduction played a key role in regulating the plant defense by stimulating the various signaling pathways. We identified a number of genes belonging to receptor protein kinases/receptor-like protein kinases. Most of the PRRs characterized so far are receptor like kinases (RLKs; Dmochowska-Boguta et al., 2015). Among this cysteine-rich receptor-like protein kinases were most up-regulated followed by wall-associated receptor kinase 2 and lectin-domain containing receptor kinases. In barley regulation of basal resistance has been reported by a powdery mildew-induced cysteine-rich receptor-like protein kinase (Rayapuram et al., 2012). Cysteine-rich receptor-like protein kinases were found to be the most likely candidate genes for powdery mildew resistance in cucumber by Xu et al. (2016). There are number of reports indicating the direct involvement of wall-associated kinases in signaling in the plant response to pathogen infection. In rice, *OsWAK1* was found to be induced by *Magnaporthe oryzae* and played a crucial role in resistance against rice blast (Li et al., 2009). Dmochowska-Boguta et al. (2015) identified three wheat WAKs strongly induced by leaf rust pathogen in wheat lines differing for leaf rust resistance genes. Lectin-domain containing receptor kinase contains extracellular motifs that are known to bind various carbohydrates; they may be involved in recognition of pathogen specific carbohydrate moieties. Lectin receptor kinases are also emerging as potential components and regulators of PRR complexes (Macho and Zipfel, 2014).

Pathogenesis related proteins such as chitinases and β-1,3-glucanases are two important hydrolytic enzymes that are abundant in many plant species after infection by different type of pathogens. The amount of these proteins significantly increase and play main role of defense reaction against fungal pathogen by degrading cell wall, because chitin and β-1,3-glucan is also a major structural component of the cell walls of many pathogenic fungi. β-1,3- glucanases appear to be coordinately expressed along with chitinases after fungal infection. This co-induction of the two hydrolytic enzymes has been described in many plant species, including pea, bean, tomato, tobacco, maize, soybean, potato, and wheat (Ebrahim et al., 2011). Overexpressed chitinases and glucanases were more abundant in WL711+*Lr57* than the susceptible parental genotype and these appeared to be induced immediately after infection with leaf rust pathogen as most of these transcripts showed upregulation in the 0–12 HPI interval. Although some transcripts continued to show upregulation in subsequent time points. An increase in chitinase and glucanase gene expression has been observed upon infection with stripe and leaf rust pathogen by Casassola et al. (2015) and Dobon et al. (2016), respectively. Pathogenesis related transcripts with Barwin, CAPS and Bet_v_1 domain were identified only in the resistant genotype WL711+*Lr57* indicating a direct link to *Lr57*. Pathogenesis related proteins have been detected to have antifungal activities in different plant systems including wheat, rice, pepper, lentil (Hwang et al., 2014). PR4 category of proteins have been found to have ribonuclease and DNAse activities as well. Pepper pathogenesis protein PR4b has been reported to even interact with NLR protein LRR1 (Hwang et al., 2014). The accumulation of PR protein is one of the best-characterized plant defense responses. In monocots, several homologs of the dicot PR genes have been identified. PR gene expression has also been observed in wheat seedlings infected with *Blumeria graminis*. In particular, PR1 and PR2 homologs have been characterized in wheat and found to be induced in both incompatible and compatible interactions with *B. graminis* (Molina et al., 1999). Manickavelu et al. (2010) observed PR1.2 protein expression only in susceptible or compatible reaction showing activation of the systemic acquired response to infection by leaf rust but in the present study PR gene expression was observed only in the incompatible reaction scenario. PR proteins have also been implicated in leaf rust resistance in genotype Toropi by Casassola et al. (2015) and proposed to be involved in restricting secondary hyphal growth. Expression of PR genes with similar functions have been proposed to be triggered by different stages of *P. triticina* development.

A number of domains observed to be involved in resistance mechanism in plants were also observed to be differentially expressed in the present RNAseq data set such as Bowman-Birk type proteinase inhibitor (BBI) a class of plant serine protease inhibitors are thought to play an important role in inhibiting trypsin and chymotrypsin of external origin (Qu et al., 2003). ABC2_membrane domain, also known as transporters are involved in detoxification processes, response to abiotic stresses and pathogen resistance (Kang et al., 2011) were differentially expressed in both the genotypes. They are involved in the above ground and below-ground secretion of secondary compounds which form an important first line of defense against pathogens (Kang et al., 2011). Oxalate oxidases produce hydrogen peroxide for developmental and stress-related response in the apoplast and may play an important role in several aspects of plant growth and defense mechanisms (Berna and Bernier, 1999). At the time of invasion fungal pathogens produce millimolar concentrations of oxalic acid for etching the cell surface of plants and interfering with guard cell function (Stotz and Guimaraes, 2004). Plant cells express oxalate oxidase as an important defense against fungal attack by eliminating the oxalic acid and producing hydrogen peroxide, which can act as a fungicidal agent and signal transduction for plant defenses and development (Pan et al., 2007). O-methyltransferase (OMT) are generally involved either in formation of precursors of lignin or in synthesis of phytoalexins and play roles in plant pathogen interaction (Manickavelu et al., 2010). Caffeoyl-o-methyltransferase (involved in the responding to wounding or pathogen challenge by the increased formation of cell wall-bound ferulic acid polymers) and jasmonate o-methyltransferase (catalyzes the methylation of jasmonate into methyl jasmonate, a plant volatile that acts as an important cellular regulator mediating diverse developmental processes and defense responses) were differentially expressed at 0–12 HPI in WL711+*Lr57* where their expression was 2.9- and 5.5-fold, respectively. Phenylalanine ammonia-lyase (PAL) participate in secondary phenylpropanoid metabolism and biosynthesis of salicylic acid (SA) which is essential for plant systemic resistance (Mauchmani and Slusarenko, 1996; Nugroho et al., 2002; Chaman et al., 2003). Plant glutathione S-transferase is known to be involved in various stress responses such as pathogen attack, oxidative stress and heavy-metal toxicity (Marrs, 1996). Multiple genes of chlorophyll a/b binding proteins were down regulated in susceptible as well as resistant interaction across all time intervals after infection thus reducing the rate of photosynthesis in order to limit the food source i.e., sugar production for invading pathogen. It was also observed in a rice–rice blast fungus interaction that transcription of photosynthetic genes, such as ribulose 1,5-diphosphate carboxylase and chlorophyll a/b-binding genes, was suppressed in both the susceptible and resistant interaction (Jantasuriyarat et al., 2005).

WL711+*Lr57* showed the occurrences of defense related genes and most prevalent categories were cellular, metabolic and biosynthetic processes. A variety of response related processes such as responses to stress, abiotic stimuli, biotic stimuli, external stimuli, endogenous stimuli, extracellular stimuli, and processes related to cell communication, cell death, abscission, and secondary metabolic processes were differentially expressed. Occurrence of such diverse stimuli elucidated the cross talk between different stress response mechanisms. While the WL711 showed the majority of processes related to regulation of transcription DNA-dependent, oxidation-reduction process and photosynthesis light harvesting. Other defense related processes were defense response to fungus, response to salt stress, response to stress, defense response to bacteria with involvement of lesser genes/transcripts. Less response elements were expressed in susceptible genotype showing that the WL711+*Lr57* have much more factors creating an effective resistance mechanism. Molecular functions showed binding, catalytic activity, hydrolase activity as prominent functions in WL711+*Lr57* while in WL711, it consisted of metal ion binding, ATP binding, DNA binding. Gene co-expression network analysis using WGCNA showed occurrence of different gene clusters in both genotypes demonstrating the differential behavior of genes at different time intervals. Each cluster centered on transcription factor except the eighth cluster of WL711+*Lr57*. The highest occupied clusters contained most of the R-genes, WL711 contained equally up and down regulated genes while WL711+*Lr57* contained mostly up-regulated elements in the highest occupied cluster 1, cluster 2 in WL711+*Lr57* consisted of mostly down-regulated genes, cluster 4 consisted of genes showing continued differential expression after 12 HPI, cluster 8 consisted of mostly down-regulated genes. These gene groups gave an idea about how cellular machinery worked during the infection.

Overall, RNAseq at different time points in resistant and susceptible genotypes after infection led to the identification of differentially expressed transcripts. It led to the identification of some transcripts which were specifically expressed only in WL711+*Lr57*. These candidate transcripts included R genes, PR proteins, peroxidases and transcription factors. Further detailed analysis of such and other identified genes with major differences in expression pattern in resistant and susceptible genotype should provide new information about mechanism of resistance conferred by *Lr57*.

## Conclusions

Whole transcriptome profiling of leaf rust resistance gene *Lr57* after challenging with leaf rust led to the identification of more than 2700 differentially expressed transcripts in WL711+ *Lr57*. A sudden burst of differential gene expression was observed at initial stages after infection i.e., 12 HPI in both WL711+ *Lr57* and parental genotype. Differentially expressed transcripts with NB-ARC and LRR domains and a number of other genes involved in plant resistance such as chitinases, glucanases, and PR proteins were more abundant in WL711+*Lr57* than in WL711. An abrupt change in expression of transcription factors after inoculation was evident in both the genotypes. Signal transduction played a key role in regulating the plant defense by stimulating the various signaling pathways. A number of genes belonging to receptor protein kinases/receptor-like protein kinases were identified. A total of 13 KEGG pathways were classified with carbohydrate metabolism as most prominent. Gene co-expression network analysis showed occurrence of different gene clusters in both genotypes demonstrating the differential behavior of genes at different time intervals with each cluster centered on a transcription factor. Highest occupied clusters contained most of the NLR-genes and resistant genotype WL711+*Lr57* contained mostly up-regulated elements in the most of the gene clusters. Overall, a number of genes specific for the genotype carrying *Lr57* have been identified which will be used for validation.

## Data submission

Total RNAseq data have been deposited at National Center for Biotechnology Information (NCBI) in Short Read Archive (SRA) database under the accession number SRP078210 and the assembled transcripts were deposited as Transcriptome Shotgun Assembly project at GenBank under the accession GEWU00000000.

## Author contributions

IY and AS carried out the RNA sequence data analysis and helped in the draft of the manuscript. SK conducted the rust inoculations and maintained the NILs used for the present study. NN helped in the preparation of the transcriptome assembly. SB supplied the rust inoculum and helped in the inoculation for time course experiment. TS and PC conceived the idea, designed and supervised the study, prepared the draft of the manuscript and submitted it. All the authors have read the manuscript and approve it.

### Conflict of interest statement

The authors declare that the research was conducted in the absence of any commercial or financial relationships that could be construed as a potential conflict of interest.

## Supplementary material

Figure S1 **Identification of correlated genes clusters using WGCNA**. In the hierarchical dendrogram, lower branches correspond to higher co-expression (height = Euclidean distance). **(A)** Four gene modules identified in WL711. **(B)** Eight modules identified in WL711+*Lr*57.

Figure S2 **Sequence alignment of potential *Lr57* candidate sequence in WL711 and WL711+*Lr57***. Candidate *Lr*57 reads from WL711+*Lr*57 were extracted and assembled into transcript. The assembled sequence was aligned with the reference sequence in WL711. Sequence alignment identified 6bp indel and 42 single nucleotide polymorphisms.

Table S1 **Summary of the Gene Ontology enrichment in WL711+Lr57 and WL711**.

Table S2 **Detailed list of the differentailly expressed R genes in WL711+Lr57 and WL711**.

Table S3 **Detailed list of the differentailly expressed genes related to transcription factors in WL711+Lr57 and WL711**.

Table S4 **Detailed list of the differentailly expressed pkinases in WL711+Lr57 and WL711**.

Table S5 **Detailed list of the differentailly expressed chitinases, glucanases and PR proteins in WL711+Lr57 and WL711**.

Table S6 **Detailed list of the differentailly expressed genes related to different domains involved in resistance in WL711+Lr57 and WL711**.

## Abbreviations

DEG: differentially expressed genes
RNAseq: RNA sequencing
NLR: nucleotide binding leucine rich repeat
HMC: haustorium mother cell
SSV: substomatal vesicles
HPI: hours post inoculation
GO terms: Gene ontology terms
tid: transcript ID
PRR: Pattern recognition receptors
PAMP: pathogen-associated molecular patterns
PTI: pattern-triggered immunity
ETI: Effecter-triggered immunity
WGCNA: Weighted Gene Co-Expression Network Analysis
NB-LRR: nucleotide binding leucine rich repeat
GH19: Glycoside hydrolase family 19
GH18: Glycosyl hydrolases family 18
GH17: Glycosyl hydrolases family 17
GH16: Glycosyl hydrolases family 16.
