# Supplementary figures and images for: Comparative Temporal Transcriptome Profiling of Wheat near Isogenic Line Carrying Lr57 under Compatible and Incompatible Interactions
Source: Front Plant Sci. 2016 Dec 23;7:1943. doi: 10.3389/fpls.2016.01943 (PMC5179980; doi:10.3389/fpls.2016.01943)

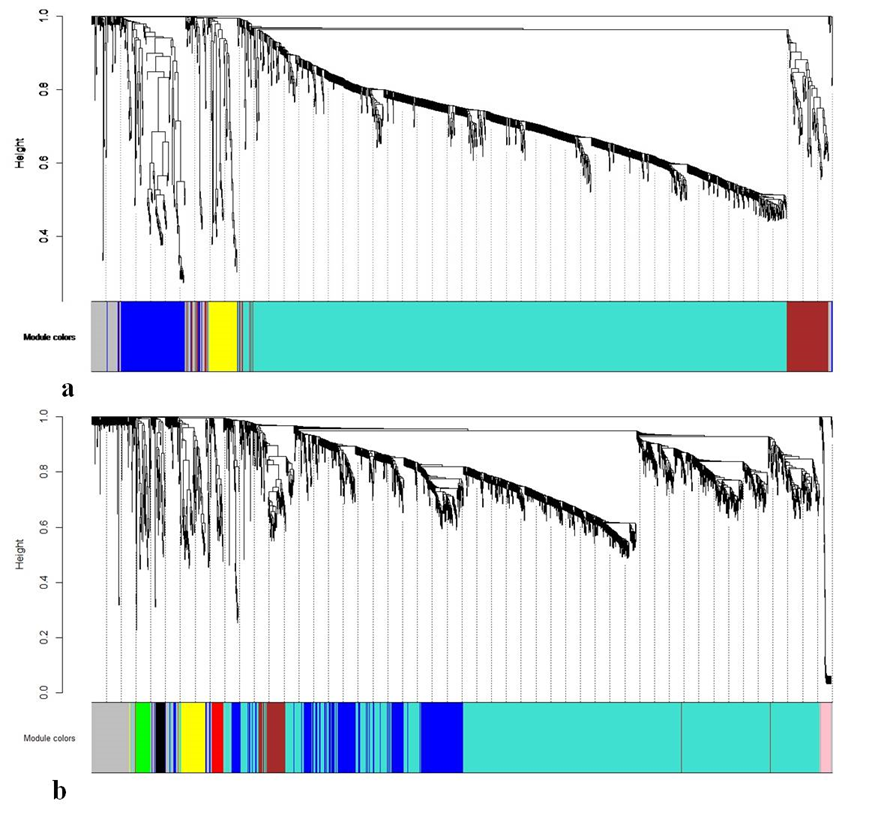

Supplement: Figure S1 — Identification of correlated genes clusters using WGCNA. In the hierarchical dendrogram, lower branches correspond to higher co-expression (height = Euclidean distance). (A) Four gene modules identified in WL711. (B) Eight modules identified in WL711+Lr57. [file Image1.TIF]

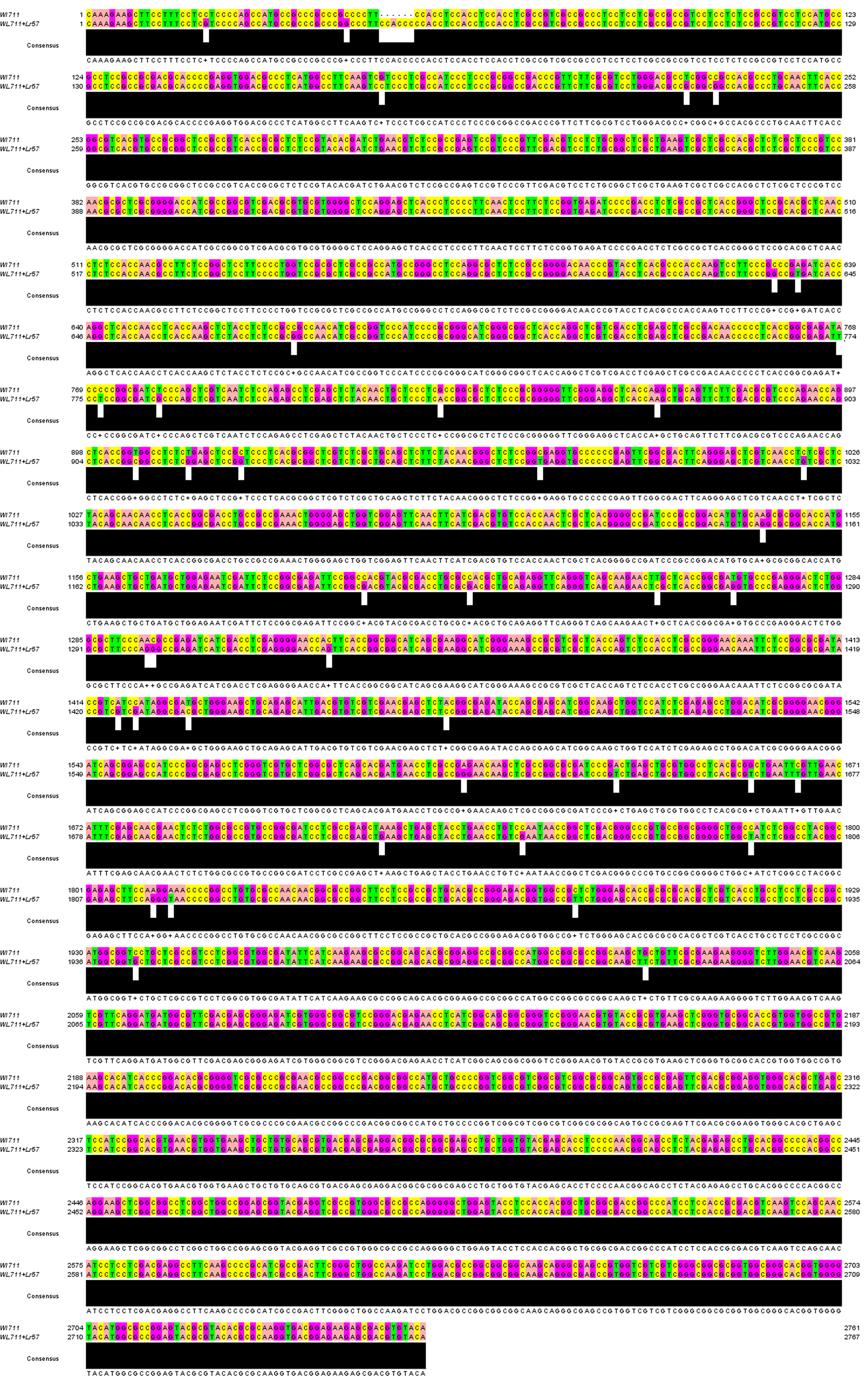

Supplement: Figure S2 — Sequence alignment of potential Lr57 candidate sequence in WL711 and WL711+Lr57. Candidate Lr57 reads from WL711+Lr57 were extracted and assembled into transcript. The assembled sequence was aligned with the reference sequence in WL711. Sequence alignment identified 6bp indel and 42 single nucleotide polymorphisms. [file Image2.TIF]
